# Predicting the murine enterocyte metabolic response to diets that differ in lipid and carbohydrate composition

**DOI:** 10.1038/s41598-017-07350-1

**Published:** 2017-08-18

**Authors:** Neeraj Sinha, Maria Suarez-Diez, Evert M. van Schothorst, Jaap Keijer, Vitor A. P. Martins dos Santos, Guido J. E. J. Hooiveld

**Affiliations:** 1 Nutrition, Metabolism & Genomics Group, Division of Human Nutrition, Wageningen University, Stippeneng 4, Wageningen, 6708 WE The Netherlands; 2 Laboratory of Systems and Synthetic Biology, Wageningen University, Stippeneng 4, Wageningen, 6708 WE The Netherlands; 3 Human and Animal Physiology, Wageningen University, De Elst 1, Wageningen, 6708 WD The Netherlands; 4 LifeGlimmer GmbH., Markelstrasse 38, Berlin, 12163 Germany

## Abstract

The small intestine serves as gatekeeper at the interface between body and diet and is thought to play an important role in the etiology of obesity and associated metabolic disorders. A computational modelling approach was used to improve our understanding of the metabolic responses of epithelial cells to different diets. A constraint based, mouse-specific enterocyte metabolic model (named *mmu_ENT717)* was constructed to describe the impact of four fully characterized semi-purified diets, that differed in lipid and carbohydrate composition, on uptake, metabolism, as well as secretion of carbohydrates and lipids. Our simulation results predicted luminal sodium as a limiting factor for active glucose absorption; necessity of apical localization of glucose transporter GLUT2 for absorption of all glucose in the postprandial state; potential for gluconeogenesis in enterocytes; and the requirement of oxygen for the formation of endogenous cholesterol needed for chylomicron formation under luminal cholesterol-free conditions. In addition, for a number of enzymopathies related to intestinal carbohydrate and lipid metabolism it was found that their effects might be ameliorated through dietary interventions. In conclusion, our improved enterocyte-specific model was shown to be a suitable platform to study effects of dietary interventions on enterocyte metabolism, and provided novel and deeper insights into enterocyte metabolism.

## Introduction

Obesity and related disorders, such as cardiovascular diseases, insulin resistance and type 2 diabetes, have become major public health issues^1, 2^, and suboptimal nutrition is among the leading preventable causes^3, 4^. The average diet in Western countries, such as the United States and the Netherlands, provides approximately 16%, 48%, and 33% of total energy intake in the form of protein, carbohydrate and lipid, respectively^5, 6^. Upon ingestion, the three macronutrients i.e. carbohydrates, lipids and proteins are broken down in the gastrointestinal tract to their basic units (monosaccharides, fatty acids and amino acids), which in turn are absorbed by the enterocytes. These monomers are subsequently used as substrates for cellular energy production, interconversions or stored for shorter or longer time period not only in enterocytes but also in other body cells via shuttling into the circulation.

After ingestion, dietary di- and polysaccharides are degraded into monosaccharides that can be absorbed^7^. It is well accepted that glucose and galactose are transported across the brush border membrane into the enterocyte by the Na^+^ dependent glucose cotransporter 1 (SGLT1). SGLT1 has high affinity for these substrates and uptake is driven by the Na^+^ electrochemical gradient across the brush border membrane that is maintained through the basolateral Na^+^/K^+^ ATPase pump. Whether or not the facilitated transporter GLUT2 is involved in the apical glucose uptake remains controversial^8^. Fructose is absorbed at the apical side by the facilitated fructose transporter 5 (GLUT5). All three hexoses exit the enterocyte at the basolateral membrane via GLUT2, although it has been suggested that fructose may also use the basolaterally located GLUT5. These carriers deliver these monosaccharides to the capillaries and portal blood.

Triacylglycerols (TAG) represent the largest component (>90%) of the dietary lipids. TAG predominantly contain long-chain fatty acids esterified to a glycerol backbone. Hydrolysis of TAG into 2-monoacylglcyerol (MAG) and free fatty acids (FFA) occurs mainly in the duodenum^7^. FFA and MAG are subsequently either absorbed by specific carrier molecules including FATP and CD36 or diffuse into enterocytes. Since both products are potentially toxic due to their detergent-like properties, especially FFA at higher concentrations, they must be rapidly neutralized after absorption^9^. This can be achieved by binding to fatty acid–binding proteins (FABP1, FABP2), which also prevents their transport back into the intestinal lumen and facilitates their intracellular transport. FFA may also be catabolized by oxidation in the mitochondria or peroxisomes, a process involving various enzymes including CPT1, ABCD1/ALDP, ACOX1, and ACAD. However, quantitatively most important is the re-esterification of FFA with MAG by MGAT and DGAT in the endoplasmic reticulum (ER) which results in the formation of di- and triacylglycerol respectively. Alternatively, FFA are esterified with free cholesterol by ACAT. These resynthesized lipids either become part of cytosolic lipid droplets and stored, or are transported to the ER for secretion into the lymph system as chylomicrons^10^. The formation of chylomicrons occurs in the ER in two steps involving APOB-48 and MTTP. Chylomicrons consist of different types of TAG (~95%), phospholipids (~4%), cholesterol or cholesteryl esters (~1%) and proteins (~2%)^11^ and are typically synthesized during postprandial periods. During post-absorptive and fasting periods, intestinal very low-density lipoprotein (VLDLs) are synthesized that consist of TAG (~40%), phospholipids (~15%), cholesterol (~15%) and proteins (~10%)^11^. Chylomicrons as well as VLDLs leave the enterocyte by exocytosis and reach the lymphatic circulation through the lateral intercellular spaces.

Although the general metabolic functions of enterocytes are known^7^, a detailed understanding of many aspects of intestinal nutrient metabolism is lacking. Such detailed understanding is needed in view of the important role the intestine is thought to play in the development of chronic metabolic diseases^12, 13^, and to explore possibilities of developing dietary strategies to prevent this and protect against enterocyte malfunctioning. Unresolved questions remain regarding the involvement of GLUT2 in luminal glucose uptake^8^ and whether the small intestine can act as a site for gluconeogenesis^14^. In addition, interactions between carbohydrate and lipid absorption and metabolism remain largely unknown. These and other issues require a systems level approach where all the metabolic interconversions and pathways are simultaneously examined to uncover possible relationships between seemingly unrelated pathways.

A computational model able to reflect specific dietary conditions and their impact on the enterocytes provides a framework for such a holistic approach^15^. *In silico* reconstruction of metabolic networks and consequent analysis of genome-scale metabolic models through constraint-based modelling helps in investigating physiology and metabolism of any cell type. Constraint-based models have been constructed using growth phenotype data for various organisms including *Saccharomyces cerevisiae*, *Homo sapiens*, *Mus musculus*, and these models predict the response of the cell or tissues to environmental and genetic perturbation and to simulate known and hypothesized phenotypes. Constraint-based modelling aims at helping researchers to get a better insight into the complex system of metabolism^16^. One of the most common approaches to constraint-based model is the use of flux balance analysis^17–22^ which can predict optimal (maximum or minimal) flux through a selected reaction using linear programming with the knowledge of reaction stoichiometry, biomass composition and additional constraints, such as limits on uptake/excretion rates and thermodynamic constraints on reaction directionality. To this end a murine enterocyte-specific constraint-based metabolic model was constructed by extensively curating and expanding a published model of human small intestinal epithelial cells^23^. Our model was then integrated with experimental data corresponding to four precisely defined dietary interventions performed in mice to study nutrient absorption and metabolic capability of enterocytes. Finally, the integrated model was used to explore the synergies between diet and enzymopathies, and to identify dietary interventions that might alleviate the effects of enzymopathies.

## Results

Building on an already published model^23^, a murine enterocyte-specific constraint-based metabolic model was developed with dedicated pathways representing TAG absorption and metabolism. Four diets, that differed in the lipid to carbohydrate ratio, were precisely quantified in terms of their nutritional composition^24^. Dietary uptake rates were used to constrain the model of enterocyte metabolism. The model was deployed to investigate the absorption of dietary TAG and carbohydrates by enterocytes and to analyze the changes that occurred when enterocytes were exposed to different dietary carbohydrate to lipid ratios while dietary protein remained unchanged in a cholesterol free diet. Changes in absorption and secretion profiles of carbohydrates and lipids were investigated. Finally, the effects of enzymopathies were studied as well as the extent to which their effects could be counterbalanced through dedicated dietary interventions.

### Construction of a constraint-based metabolic model for murine enterocytes

**Figure 1:**
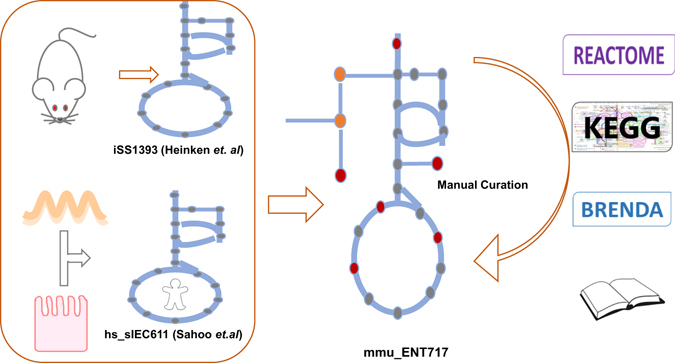
Model construction. Schematic overview of the construction process of a metabolic model of murine enterocytes. An existing genome scale murine metabolic model and a model of human small intestinal epithelial cells were combined; the resulting draft underwent extensive manual curation to arrive to *mmu_ENT717*. Key features of this model are summarized in Table 1.

To investigate the adaptability of nutrient absorption and metabolism in murine enterocytes when exposed to different diets, we required an enterocyte-specific computational framework. To this end a previously published human small intestinal epithelial cell metabolic model with 611 unique genes (*hs_sIEC611*)^23^ was first combined with mouse specific information as described in mouse reconstruction *iSS1392* ^25^. The resulting model was extensively curated and expanded, especially regarding lipid metabolism, resulting in a model comprising 708 unique metabolites that are involved in 1830 reactions and has 717 genes associated (Fig. 1). This model was named *mmu_ENT717*, where ‘*mmu*’ stand for *Mus musculus*, ‘ENT’ for enterocyte, and ‘717’ for the total number of associated genes. The model is available in Supplementary file S1-reactions and S2-metabolites. Compared to *hs_sIEC611* our model represents an important increase in the number of described reactions (>14%) and most importantly, a 63% increase in the number of metabolites accounted for (Table 1). This increase was mainly due to the complete incorporation of detailed reactions describing absorption, (re)synthesis, oxidation and secretion for all 6 fatty acid species present in the studied diets. Pathways corresponding to the synthesis of the respective cholesteryl esters and formation of chylomicrons, that were lacking in the original model *hs_sIEC611*, were explicitly curated. In addition, specific transporters involved in carbohydrate metabolism were added, including the mitochondrial pyruvate importer heterodimers MPC1 and MPC2. Since enterocytes do not proliferate, production of biomass from precursors became insignificant. Instead, the capability of the model to perform specific metabolic tasks such as fatty acid and carbohydrate metabolism, secretion of lactate from glucose uptake, among others, were examined as a measure of the metabolic activity of enterocytes and to assess model quality. Finally, the compartment of Golgi bodies was added to the model. Thus, our model is comprised of six intracellular compartments (cytoplasm, nucleus, mitochondria, peroxisomes, Golgi body, endoplasmic reticulum) and two extracellular compartments (intestinal lumen [apical], extracellular [basolateral]), which all have different (metabolic) roles. The consistency of our model was tested and no holes in the structure of the network were identified. In addition, the model was used to recapitulate known biological functions of small intestine enterocytes. These functions relate, mostly, to the secretion of selected metabolites and were the same as listed in and used by Sahoo & Thiele^23^ for the construction of their human small intestinal epithelial cell metabolic model (*hs_sIEC611*). Our model was successfully challenged to perform these (and other) tasks, and this was an important validation and quality criterion. Simulations regarding these tasks are available in Supplementary file S4-Metabolic tasks. Our comprehensive constraint-based metabolic model was next used to investigate the metabolic impact on enterocytes of four precisely defined dietary interventions. The model was seen to be able to carry flux through the reactions associated to these tasks under constraints associated to the four dietary interventions (see Supplementary file S4-Metabolic tasks). It should be noted, however, that these represent qualitative validations of the performance of the model. Quantitative validation would entail, among others, *in vivo* measurements of secretion rates. In this study, the model was used to explore avenues of nutritional physiology.

**Table 1:** Description and comparison of the published human smal﻿l intestinal﻿ ﻿(*hs_sIEC611*) and e﻿xpanded ﻿murine-specific enterocyte (*mmu_ENT717*) models.

	*hs_sIEC611*	*mmu_ENT717*
Total number of reactions	1282	1830
Transport & Metabolic Reactions	999	1148
Exchange/demand/sink/biomass reactions	267/14/1/1	262/14/1/1
Unique metabolites	433	708
Genes	611	717
Intracellular compartments	5	6

### Diets

**Figure 2:**
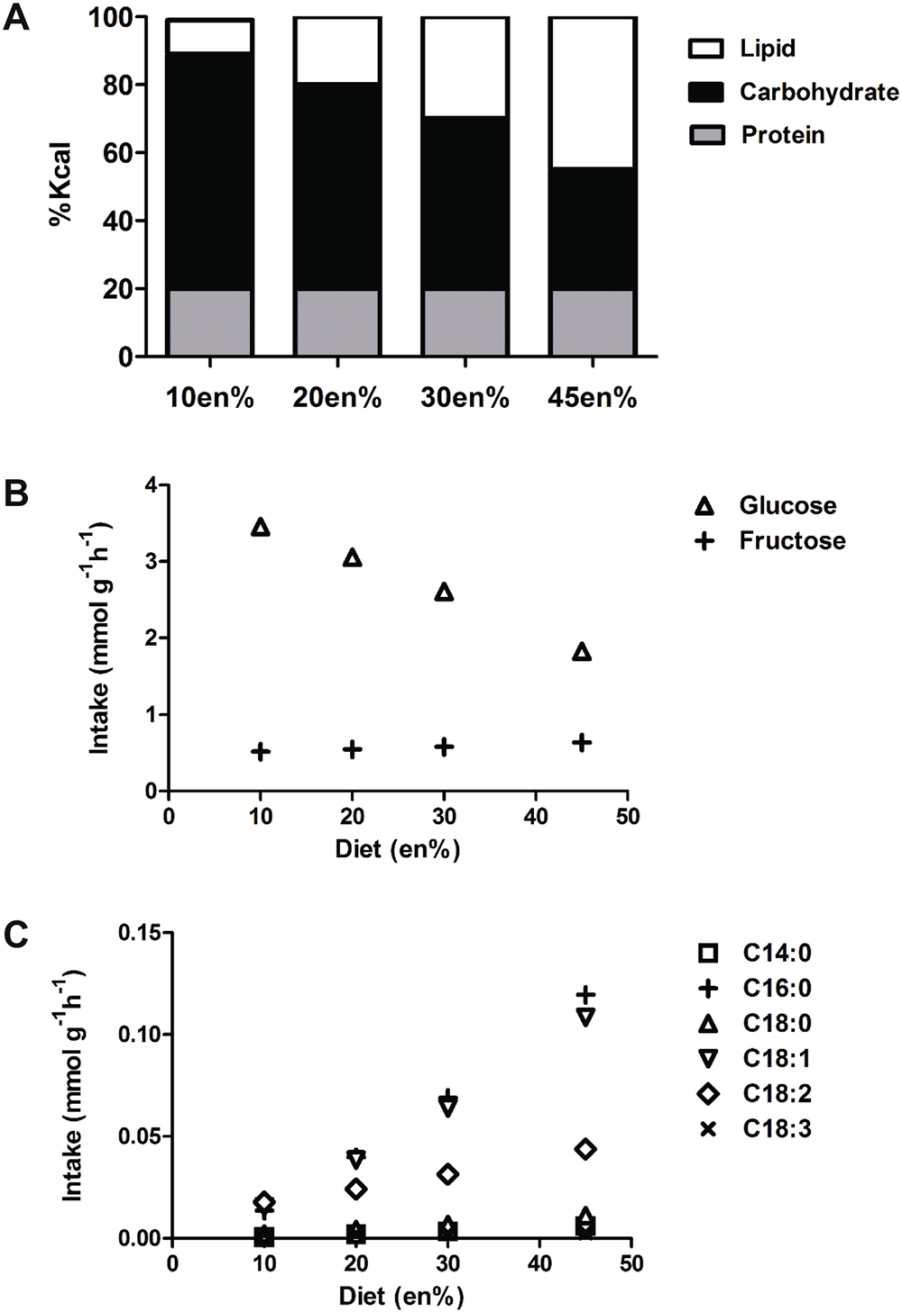
Diet description and intake. (**A**) Overall dietary composition expressed as %kcal; (**B**) Maximal potential intake rates of glucose and fructose, the two hexoses present in the diet; (**C**) Maximal potential intake rates of the different fatty acid species present in the diet.

Nutrient absorption and metabolic capability of enterocytes was investigated using the composition of 4 well-defined, semi-synthetic diets that were used previously to study the role of the small intestine during diet-induced obesity^24^. These diets were designed to differ in lipid to carbohydrate ratios (Fig. 2A). The amount of energy derived from lipids varied from 10 (lowest) to 45 (highest) energy%, and as a consequence the carbohydrate content decreased from 69 to 35 energy%. The energy derived from protein was 20 energy% for all diets. The diets were named 10en%, 20en%, 30en% and 45en% according to the fraction of energy derived from lipids. Detailed information on the diets is provided in Supplementary file S3-Diet Composition. The decrease in starch content led to a change in ratio of fructose to glucose in each of the diets, however, sucrose content was identical (Fig. 2B). Moreover, the ratio of palm versus soy oil varied between the diets because an increase in palm oil content was exchanged with starch, while soy oil content remained constant. As a result, the fatty acid profiles of the diets differed. The diets contained 6 different fatty acids that varied in amounts (Fig. 2C). The only sources of lipids in the diets were palm oil and soybean oil, and therefore the diets were free of cholesterol. The nutrient composition of each diet was combined with the daily feed intake derived from weekly measurements and expressed as mmol per gram dry weight per hour (mmol g dry weight^−1^ h^−1^). These values were used to constrain the 39 exchange reactions in the model that represent uptake of components in the diets.

### Metabolic impact of dietary interventions

#### Carbohydrate Metabolism

**Figure 3:**
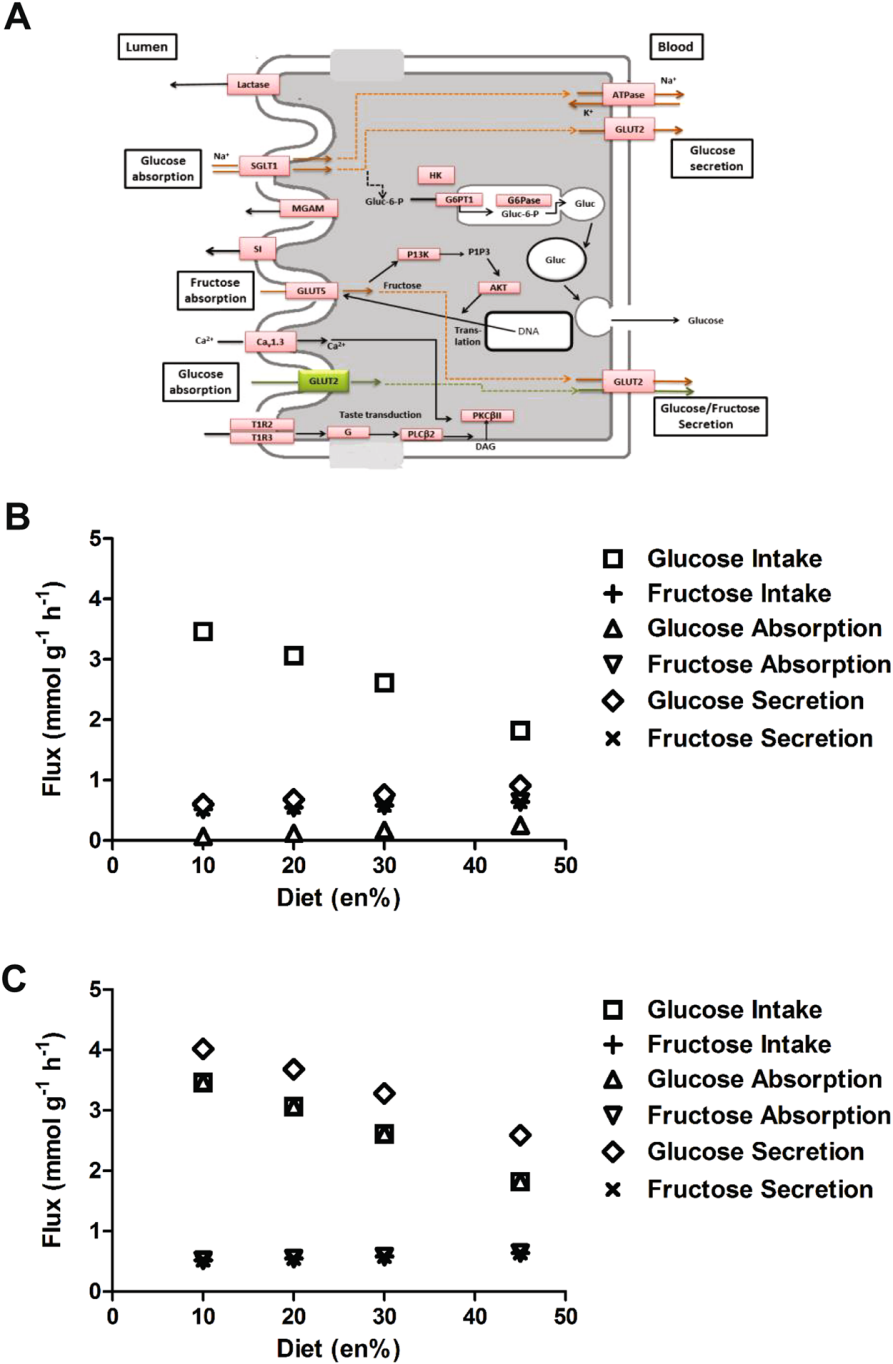
Carbohydrate metabolism in enterocytes. (**A**) Schematic overview of carbohydrate absorption and secretion pathways in murine enterocytes (adapted from KEGG). Apical and basolateral location of GLUT2 has been included in the graph; (**B**) Simulated maximal glucose and fructose absorption and secretion rates modelled in the absence of apically located GLUT2; (**C**) Simulated maximal glucose and fructose absorption and secretion rates modelled in the presence of apically located GLUT2.

**Figure 4:**
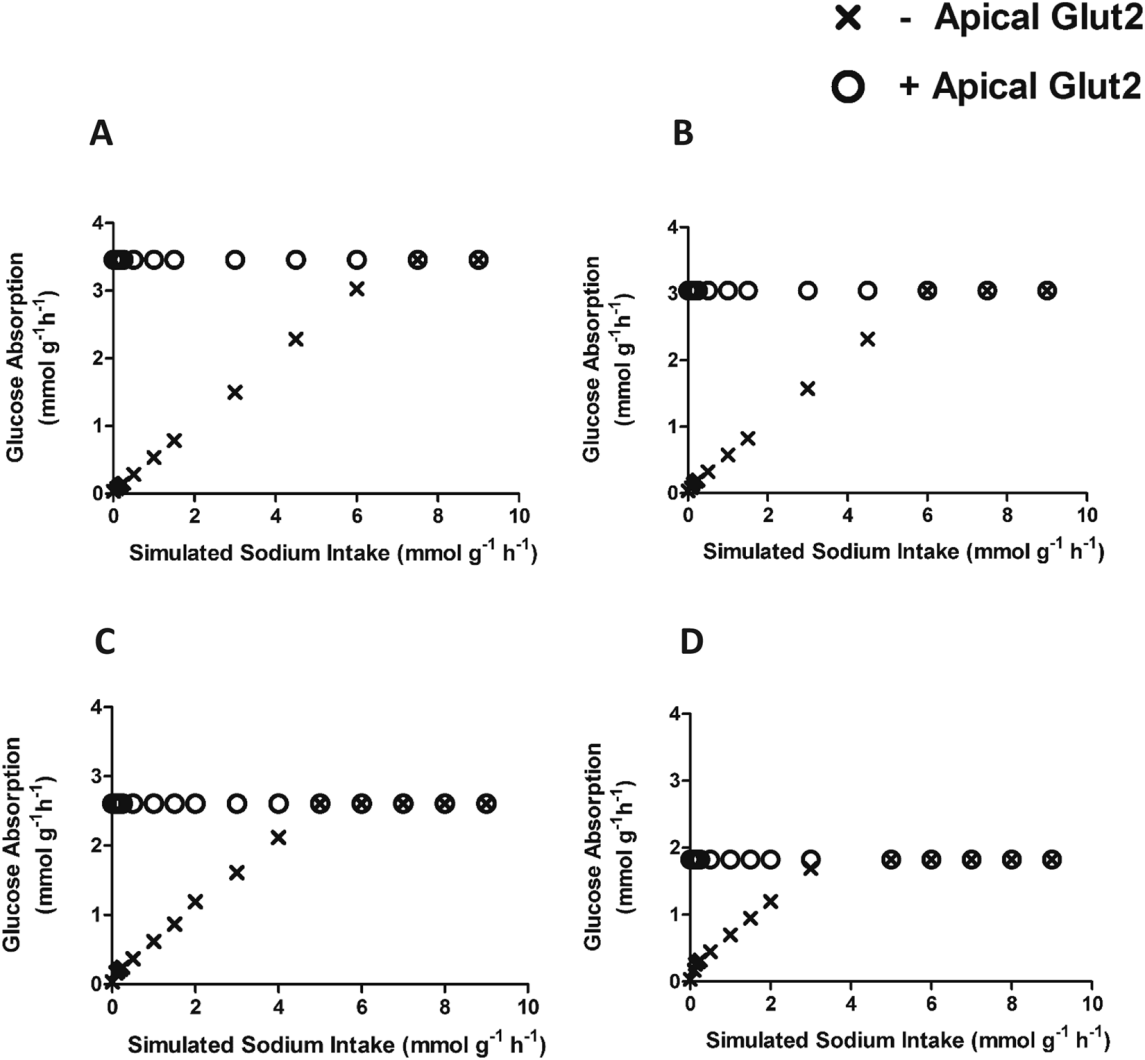
Glucose absorption *versus* simulated Na^+^ intake in the presence (+) or absence (−) of apically located GLUT2. (**A**) Simulation results for the 10 en% diet; (**B**) Simulation results for the 20 en% diet; (**C**) Simulation results for the 30 en% diet; (**D**) Simulation results for the 45 en% diet.

First focus was on the luminal absorption and portal secretion of glucose and fructose, the two monosaccharides present in the diets, because of the controversy on the role of apical GLUT2 in this process^8^. A schematic overview of the monosaccharide metabolism in enterocytes that was modelled is given in Fig. 3A. Glucose absorption in enterocytes was simulated in the presence and absence of GLUT2 on the apical side using the amounts present in the experimental diets. As objective function for each diet apical absorption and basolateral secretion of glucose was employed individually. Glucose appeared minimally absorbed in absence of apical GLUT2 (Fig. 3B), while the presence of apical GLUT2 resulted in complete absorption (Fig. 3C). Since glucose absorption via SGLT1 requires Na^+^, we used the model to compute maximal possible glucose absorption with varying Na^+^ availability in the presence and absence of apical GLUT2 (Fig. 4). These results suggested that luminal Na^+^ acts as a limiting factor for glucose absorption, but only in the absence of apical GLUT2. Moreover, the Na^+^ requirements for maximal glucose absorption in the absence of apical GLUT2 were over 10 times the amount of dietary Na^+^ (see Supplementary Table S3).

The model also predicted that for each diet the maximal possible basolateral glucose secretion rates were higher than the maximal luminal absorption rates, which pointed to the potential for gluconeogenesis, i.e. *de novo* glucose synthesis pathways, in enterocytes. This effect was even more prominent when apical GLUT2 was included in the model (Fig. 3C). The model was then used to explore the origin of this extra glucose coming into circulation. Exhaustive exploration of reactions related to gluconeogenesis, given the simulated dietary conditions, showed the origin of these gluconeogenic processes to be in amino acid related pathways. This effect was observed for all four simulated diets. In agreement with the passive absorption and secretion of fructose via GLUT5, we observed that maximal basolateral fructose secretion rates equaled its maximal apical absorption rates.

#### Lipid Metabolism

**Figure 5:**
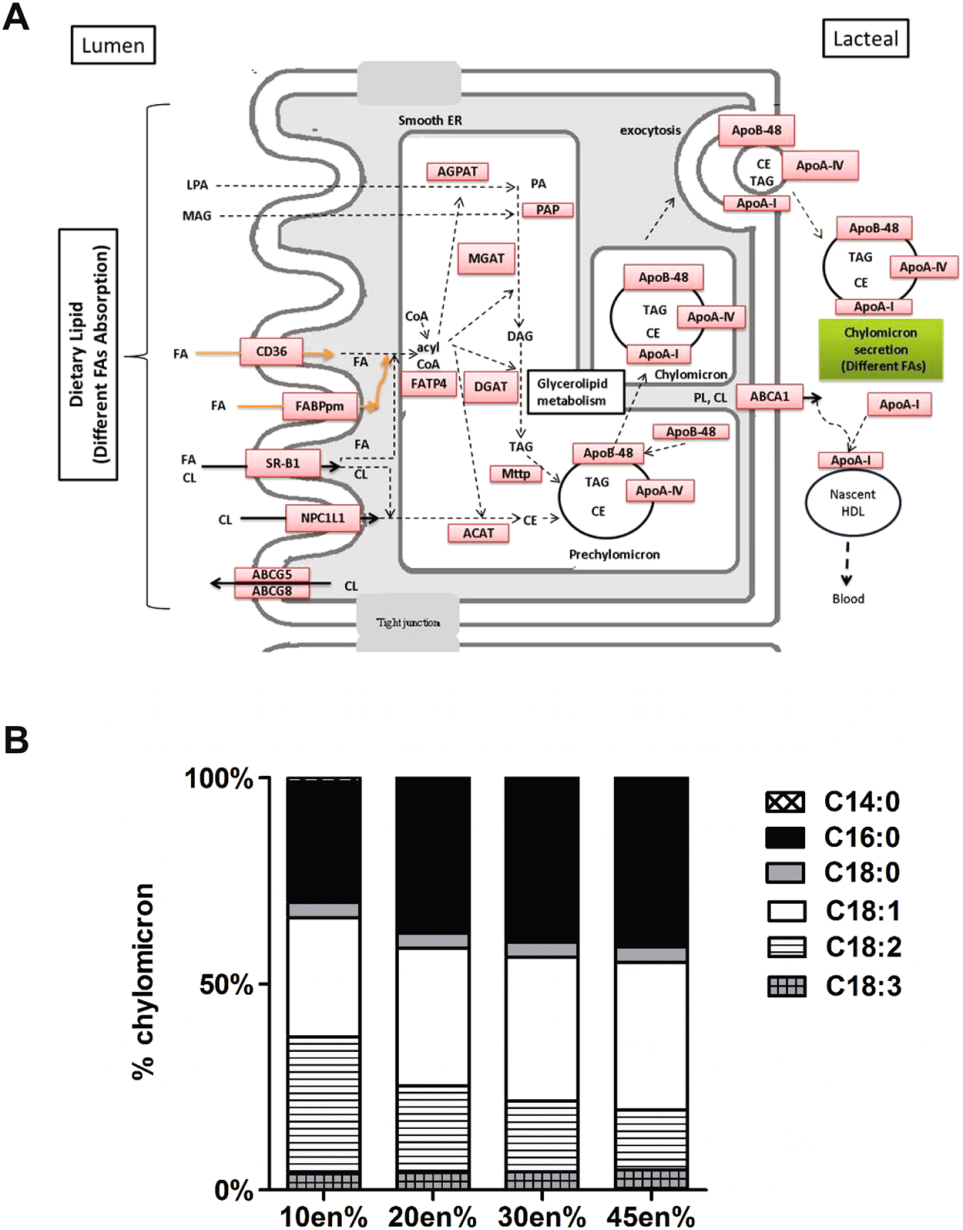
Lipid metabolism in enterocytes. (**A**) Schematic overview of lipid absorption and secretion pathways in mice (adapted from KEGG). (**B**) Fatty acid species composition of chylomicrons for the four diets.

FFA and MAG, the two hydrolysis products of dietary TAG, are absorbed from the lumen into enterocytes. In Fig. 5A a schematic overview is given of lipid metabolism in enterocytes that was modelled. As different fatty acids might have different effects on metabolism, both fatty acid composition and maximal chylomicron secretion rates were modelled for all four diets. Initial simulations in which chylomicron secretion was maximized, showed that the model was unable to simulate chylomicron formation and secretion irrespective of dietary TAG content or composition. Model inspection by iterative maximization of the flux through each reaction in the model showed that this was due to lack of available intracellular cholesterol that is required for chylomicron formation. This, in turn, could be attributed to the absence of uptake of luminal cholesterol, since the diets were lacking cholesterol and biliary cholesterol availability was not modelled. However, simulating the model using the *in silico* addition of intracellular cholesterol, reflecting luminal absorption of biliary cholesterol, indeed resulted in chylomicron secretion. Alternatively, we modelled the effect of *in silico* addition of endogenous cellular biosynthesis of cholesterol which also resulted in chylomicron secretion. Cholesterol biosynthesis requires ATP, which in turn relies on mitochondrial oxygen availability, therefore the simulations of endogenous cholesterol biosynthesis were performed by allowing basolateral oxygen uptake while maximizing chylomicron secretion. In this way, the oxygen-dependency of chylomicron formation was modelled and simulation of increased oxygen availability also resulted in elevated chylomicron secretion. Using our model the minimal oxygen requirements for maximal chylomicron secretion could be estimated, and these equaled to 1.5 × 10^−5^, 3.1 × 10^−5^, 5.1 × 10^−5^ and 8.4 × 10^−5^moles/mouse/day for the 10en%, 20en%, 30en% and 45en% fat diets, respectively. The FA composition of the chylomicrons based on each of the four diets is shown in Fig. 5B, and these reflected the dietary FA composition.

### Effect of Enzymopathies on Enterocytes metabolism

**Figure 6:**
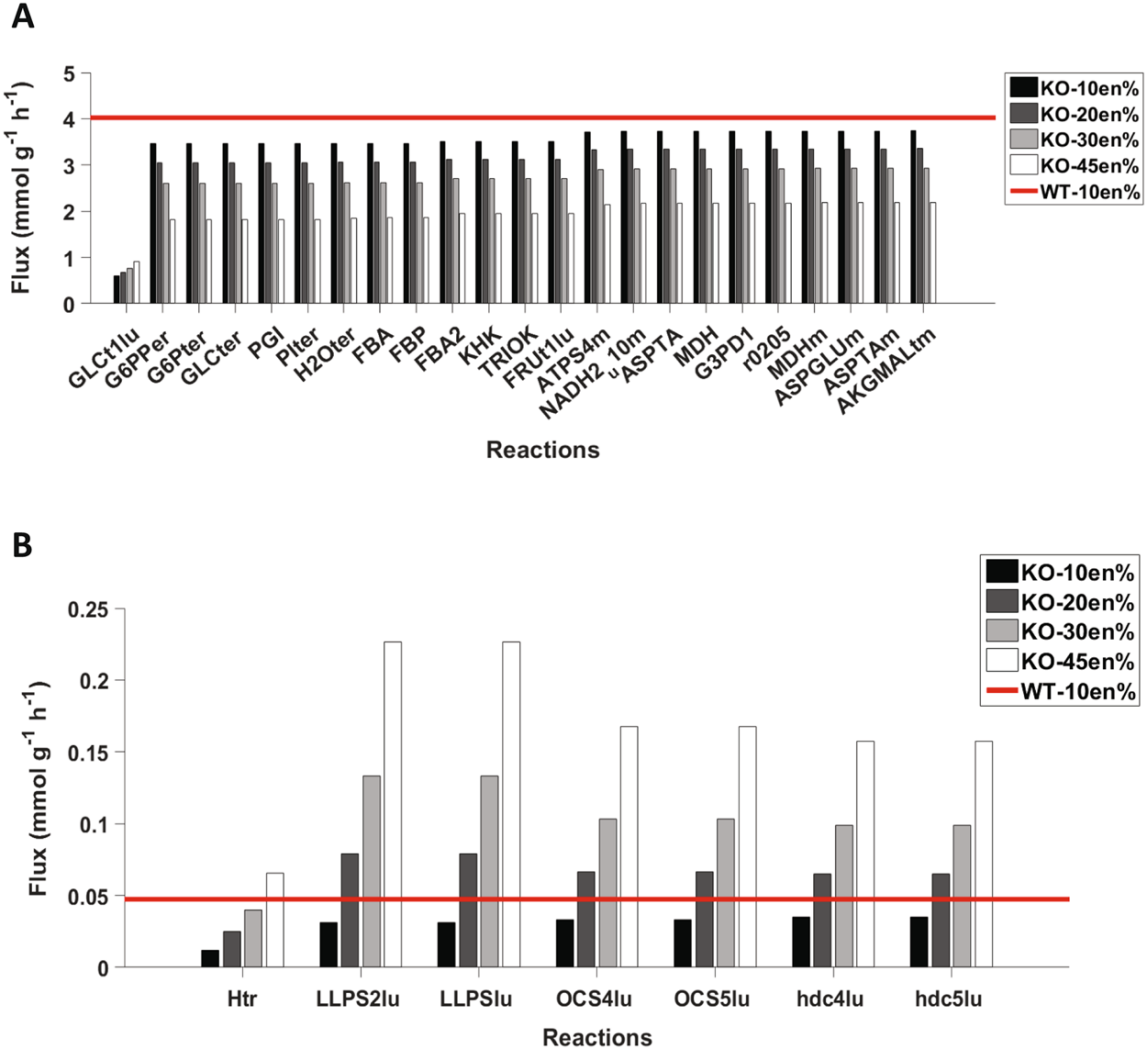
Reaction knockout for glucose and TAG. (**A**) Impact of reaction deletions on maximal glucose secretion. (**B**) Impact of reaction deletions on maximal TAG in chylomicrons secretion.

Next, our modelling approach was used firstly to identify enzyme insufficiencies that affected enterocyte carbohydrate and lipid secretion, and secondly to investigate whether the impact of these defective or absent enzymes could be alleviated or even overcome by potential dietary interventions. To this end, the maximum possible values for glucose secretion (reaction ID abbreviation GLCGLUT2) and TAG secretion as chylomicron (CHYLOMGE) were calculated for the 10en% fat diet. These values were considered as reference values and the effect of a possible impairment of each reaction present in the model was iteratively investigated. With regard to both glucose and TAG secretion, three types of behavior were observed that enabled the classification of reactions into the following three types: Type I: reactions that upon impairment did not affect the chosen task; Type II: reactions that upon impairment lead to complete blockage of the desired task; and Type III: reactions whose impairment lead to reduced performance (i.e. >5%) regarding the task but could be to some extent recovered by modifying the composition of the simulated diet. All reactions and their impact on glucose and TAG secretion are listed in Supplementary File S5-Reaction_KO. By considering maximal basolateral glucose secretion as a metabolic task and as objective function, only a single reaction of type II was identified, i.e. a reaction that upon deletion led to a complete blockage of glucose secretion, which, unsurprisingly, was GLUT2-mediated basolateral export of glucose (see Supplementary File S5-Reaction_KO_Glucose). In addition, 23 reactions were identified that upon deletion resulted in impaired, but not abolished glucose secretion as shown in Fig. 6A (Type III reactions). These Type III reactions mainly converged on glycolysis, pentose phosphate pathway, oxidative phosphorylation and amino acid transaminases. See Supplementary Figure S1A for an overview of the affected reactions on the KEGG global metabolism map). Apically-located GLUT2 location was again observed as a requirement for maximal glucose uptake and subsequent secretion. When TAG secretion was simulated by employing chylomicron secretion as objective function, 41 reactions of type II were identified, i.e. reactions that upon deletion led to a complete blockage of TAG secretion. These reactions are listed in supplementary file S5-Reaction_KO_Chylomicron. Almost all of these reactions are part of the pathways required for cholesterol biosynthesis, such as the mevalonate and steroid synthesis pathways, or closely link to these. In addition, the synthesis of acetyl-CoA by ATP citrate lyase and the reaction catalysed by microsomal triglyceride transfer protein (MTTP) were also found to be essential for TAG secretion. An overview of the affected reactions on the KEGG global metabolism map can be found in Supplementary Figure S1B. The only reactions that impaired TAG secretion (Type III) were luminal lipase reactions as shown in Fig. 6B.

## Discussion

In this study, we report on a comprehensive and improved murine enterocyte metabolic model. Compared to previous models, our *mmu_ENT717* model is ideally suited to study carbohydrate and lipid metabolism as well as trans epithelial exchange from apical to basolateral side. It contains a very extensive representation of TAG/FFA metabolic reactions in enterocytes, specifically with regards to six different types of fatty acids that were present in different amounts in the considered experimental diets containing soybean and palm oil. The lipid reactions have been added following a standardized approach, using precisely defined metabolite identifiers. This set of reactions can therefore be adapted to any other metabolic model, thereby increasing the accuracy of the model. Moreover, our approach can also be easily extended to other TAG types which would allow the study of the impact of other dietary lipid compositions.

Constraint-based metabolic models require uptake rates to produce meaningful predictions. However, such detailed biochemical characterizations of dietary interventions are often lacking and in the past, population-wise averages have been used. Instead, here we have used detailed characterizations of specific diets fully representing an experimental set up; thus, these diets accurately represent actual intake levels of individual mice. The diets used represent a broad range of lifestyles as the energy derived from fat ranges from 10en% up to 45%, the latter which in humans represents an unhealthy Western-style diet. Availability of such dietary datasets greatly expands applicability of constraint-based metabolic models and we believe that the data presented in Supplemental file S3-Diet Composition will be widely used by other researchers willing to approach murine metabolism from a systems perspective. We have used the model to gain deeper understanding of nutrient absorption by enterocytes. Our main findings include (i) luminal sodium as a limiting factor for active glucose absorption in the absence of apical GLUT2, (ii) the potential of the small intestine for gluconeogenesis both in presence and absence of apical GLUT2, (iii) the association between chylomicron secretion and cholesterol biosynthesis in luminal cholesterol-free conditions and associated oxygen requirements, and (iv) consequences and potential dietary restoration options for inborn errors of metabolism in enterocytes. These aspects will be discussed in more detail.

We used the model to confirm the traffic of GLUT2 to the apical membrane upon high luminal glucose concentrations^26^. Our model showed that in the absence of apical GLUT2, cells can only absorb all the luminal glucose provided when abundant luminal sodium is present, representing SGLT1 functionality. This is in line with recent functional findings that demonstrated a reduction in luminal sodium availability after Roux-en-Y gastric bypass surgery, which in turn impaired intestinal glucose absorption^27^. We found that for full absorption of luminal glucose by only SGLT1, Na^+^ requirements were more than 10 times the amount present in the diet. However, owing to the complex physiology of the gastrointestinal tract it should be realized that luminal Na^+^ is not only derived from dietary sources, but also, and in larger quantities, from pancreatic and biliary juices^27, 28^. Our model also revealed the potential for gluconeogenesis, which was associated with amino acid availability in the diet. Indeed, it has been suggested that gluconeogenesis from glutamine may occur in the small intestine^29^. This might also be a way to dampen the influx of amino acids into circulation, thereby controlling blood pH.

Our model also enabled computation of oxygen requirements for chylomicron secretion in the absence of biliary cholesterol levels. Of note, the model does not incorporate uptake of cholesterol from circulation at the basolateral side; thus by absence of luminal cholesterol, cellular *de novo* cholesterol biosynthesis is needed for adequate chylomicron secretion. Indeed, feeding mice a cholesterol-free diet leads to upregulation of genes encoding enzymes involved in cholesterol biosynthesis in small intestinal cells^30^, and inhibition of cholesterol biosynthesis resulted to reduced production of very low density lipoprotein (VLDL) particles by the liver^31^. Here, we have also shown that increased chylomicron synthesis/secretion is associated with an increased demand of ATP and thus oxygen in the intestine, which represents at least part of intestinal tissue oxygen consumption. It is estimated that the gastrointestinal tract contributes to about 7%-11% of standard metabolic rate in adult rat and humans, respectively^32^.

It was found that in most cases the modelled fluxes were proportional to the dietary carbohydrate or lipid content (Figs 3 and 5, respectively). This implies that in the simulated scenarios no change in active constraints occur. Nevertheless, it should be noticed that no additional constraints have been imposed in the model to simulate limitations in enzyme availability or enzyme saturation characteristics. A more detailed view of these effects could be obtained by integrating the model here presented with experimental data characterizing enzyme expression and activity.

We have also explored the potential effect of dietary interventions to mitigate the effect of some metabolic enzymopathies, with emphasis on carbohydrate and lipid secretion. Most enzyme deletions did not impact secretion rates, but in few cases it was found that their effects could be reverted of partly ameliorated by an increased dietary lipid uptake. However, caution should be warranted when designing this type of interventions as intake of increasing energy percent of fat in the diet easily leads to a positive energy balance which could ultimately result in development of obesity-related metabolic diseases like type 2 diabetes and cardiovascular diseases.

In conclusion, our model accurately represents nutrient absorption of small intestinal enterocytes and represents an optimal starting point for the development of a multi-tissue and multi-scale model able to account for the function of the intestine in its entirety.

## Materials and Methods

### Construction of the murine enterocyte metabolic model

The previously published human sIEC model (*hs_sIEC611*)^23^ was improved by performing a thorough search of the current literature and various enzyme and pathway databases (BRENDA^33^, KEGG^34^, Reactome^35^, WikiPathways^36^, Recon2^37^) to obtain reactions known to occur in enterocytes, and reactions that were missing were added. Murine homologues of human genes were retrieved from the Homologene database^38^. Gene-protein-reaction (GPR) relationships associated with reactions were defined by the Boolean rules ‘*and*’ and ‘*or*’, where ‘*and*’ represents that all gene products are required while ‘*or*’ represent requirement of any of the listed gene products to catalyze the corresponding reaction. The model was, in particular, expanded with respect to lipid metabolism. In the human sIEC model the different triacylglycerols (TAG) are grouped as a single compound (R_total) that reflects the total amount of dietary TAG present. In our model we broke down the dietary TAG into 6 individual fatty acid species (myristic acid [C14:0], palmitic acid [C16:0], stearic acid [C18:0], oleic acid [C18:1], linoleic acid [C18:2], linolenic [C18:3]) and their corresponding diacylglycerols (DAG) and 2-monoacylglcyerols (MAG), since these metabolites represent the full fatty acid profile of the experimental diets given to the mice^24^. In addition, the human sIEC model was expanded with reactions for absorption, (re)synthesis, oxidation and secretion for each FA species, which resulted in the addition of 18 reactions for lipid absorption, 36 reactions for *de novo* synthesis of MAG, DAG and TAG, and 6 reactions for FA esterification with cholesterol. All unspecific reactions in the sIEC611 model that did not differentiate between the FA species were substituted by specific reactions. All different TAG were formulated based on a uniform fatty acid profile; that is all TAG molecules were composed of 3 identical fatty acid species. This was done for reasons of simplicity, because the possible number of different TAG molecules including enantiomers is *n* ^3^, where *n* is the number of fatty acids present. This number will be very large even when a limited number of fatty acids is considered^11^. Taking into account the fact that before being absorbed, the TAG molecules have to be hydrolyzed by luminal lipases resulting in the formation of the building blocks of TAG, i.e. free fatty acids and glycerol, we believe this simplification is warranted. Our final *mmu_ENT717* model is comprised of a total of 1830 reactions and 717 metabolic genes. 843 reactions had no gene associated of which 164 are diffusion reactions, whereas the remaining 565 reactions are orphan reactions. Information from the KEGG database and mouse whole genome model^25^ was used to determine candidate reactions to fill gaps in the non-compartmentalized draft model. The gap filling method from the COBRA toolbox^39^ was set to run 40 iterations. Each solution was then manually checked for applicability, such as the feasibility of the suggested directionality, before choosing a proposed solution. The Cobra model *mmu_ENT717* model is available in. *mat* format as supplemental file 3.

### Exchange reactions for dietary components to constrain the model

The composition of the diets and daily feed intake calculated from weekly intake levels has been reported previously^24^. All dietary constituents were broken down to their basic units (i.e. TG species, monosaccharides, amino acids, vitamins, minerals). The fatty acid profiles of the dietary oils (i.e. palm and soybean oils) that were used in our study were provided by the supplier, and were in very close agreement with the values reported by the worldwide food standards commissions (Codex Alimentarius, IUPAC-IUNS)^40^. Casein (milk protein) was used as the sole protein source of the diet. Amino acid composition of the casein was derived from Pennings *et al*.^41^. Uptake rates of all these 39 nutrients were then converted into moles mouse^−1^ day^−1^ (Supplementary file S3-Diet Composition). Next, the enterocytes mass per mouse, expressed as g dry weight (gdw), was calculated. Since mouse-specific data is unavailable, and the anatomy and histology of the mouse small intestine is similar to that of humans^42^, human data was used for the percentage of enterocytes of total cells in the small intestine (i.e. 55.2%)^23, 43^. Such an approach is not uncommon as for example the parametrization of the virtual physiological rat and mouse heart models include data from other species as well^44, 45^. The average wet weight of the mouse small intestine was reported to be 1.094 g, of which 76% was water^46^. The dry weight (dw) of the enterocytes per mouse equaled thus 1.094 g × 0.24 × 0.552 = 0.1449 g. The factor, A, to convert the intake of the nutrients from moles mouse^−1^ day^−1^ to mmol (gdw enterocytes)^−1^ h^−1^ is thus ${\rm{A}}=\frac{{{\rm{10}}}^{3}\,{\rm{mmol}}}{{\rm{0.1449}}\,g\,\times {\rm{24}}\,{\rm{h}}}={\rm{2.8749}}\times {{\rm{10}}}^{2}$.

### Flux balance analysis

In a constraint-based metabolic model, reactions are represented by the stoichiometric matrix (*S*) of size *m × n* (where ‘*m*’ represents the number of metabolites and ‘*n*’ represents the number of reactions in the network). In this way *S*_*ij*_ is the stoichiometric coefficient of metabolite *i* in reaction *j;* positive and negative *S*_*ij*_ values indicating metabolite production or consumption respectively. Under steady state conditions the sum of input fluxes equals the sum of output fluxes for metabolite concentration (*x*) over time (*t*) i.e. *dx/dt* = 0, so the steady state equation can be written as $S\times \vec{v}=0$ where $\vec{v}$ is a flux vector of size *n* × 1 which represent the flux through all reactions. Flux values (*v*_*i*_) are limited with a set of constraints that indicate reaction reversibility: *l*_*i*_ ≤ *v*_*i*_ ≤ *u*_*i*_ for *i* ∈ {1, …, n}. The vectors $\vec{l}$ and $\vec{u}$ contain minimum and maximum flux values for each reaction. Reversible reactions are indicated by negative *l*_*i*_ values. Additionally, *l*_*i*_ and *u*_*i*_ values can be further constrained by experimentally determined values. Reactions representing luminal or basolateral uptake or secretion rates are termed exchange reactions. The usual convention of writing exchange reactions was followed in such a way that uptake and secretion are represented by negative and positive flux values, respectively. Here we have imposed the constraints derived from the analysis of the dietary intervention to the exchange reactions present in the metabolic model to mimic the specific conditions. Specifically, (negative) uptake rates of the dietary nutrients were set as lower bounds of the exchange reactions representing luminal uptake of the corresponding components. Flux balance analysis (FBA) seeks to optimize (by either maximizing or minimizing)^23^ a given objective function under a specific condition. In this study, metabolic tasks were defined for glucose and lipids on absorption from apical and secretion at basolateral side of the cell, for all four dietary conditions. To identify the effects on metabolism of enterocytes after deletion of a reaction, we used the cobra function to delete model reaction and optimize the corresponding objective^39^. All model calculations were done in MATLAB version 2014b (The Mathworks Inc.) using the COBRA toolbox and Gurobi solver version 6^39, 47^.

## Electronic supplementary material

Supplementary information

Supplementary dataset 1

Supplementary dataset 2
