# Supplementary material for: Predicting the murine enterocyte metabolic response to diets that differ in lipid and carbohydrate composition
Source: Sci Rep. 2017 Aug 18;7:8784. doi: 10.1038/s41598-017-07350-1 (PMC5562867; doi:10.1038/s41598-017-07350-1)

Supplementary information

Predicting the murine enterocyte metabolic response to diets that differ in lipid and carbohydrate composition

Neeraj Sinha[1,2,3], Maria Suarez-Diez[2], Evert M. van Schothorst[3], Jaap Keijer[3], Vitor Martin dos Santos[2,4], and Guido J. E.J. Hooiveld[1]

[1]Nutrition, Metabolism & Genomics Group, Division of Human Nutrition [2]Laboratory of Systems and Synthetic Biology, [3]Human and Animal Physiology, Wageningen University, Wageningen, The Netherlands, and [4]LifeGlimmer GmbH, Germany

Contents:

Supplementary Dataset 1: Separate Excel file containing:

Sheet 1: Reaction information for 1830 reactions represented in model *mmu_ENT717*.
Sheet 2: Metabolite information for 708 metabolites represented in model *mmu_ENT717*.
Sheet 3: Dietary composition broken down into 39 components.
Sheet 4: Simulation results for selected metabolic task for enterocytes and flux through other reactions in the presence or absence of apical localized GLUT2.
Sheet 5: Simulation results of impact of reaction deletions on maximal glucose secretion.
Sheet 6: Simulation results of impact of reaction deletions on maximal TAG secretion in chylomicrons.

Supplementary Dataset 2: Separate ZIP archive containing:

Model *mmu_ENT717* in *mat* format.

Supplementary Figures:

KEGG global metabolism map showing the reactions that upon deletion impaired glucose secretion (Figure S1), or abolished TAG secretion (Figure S2).

Supplementary figure S1: KEGG global metabolism map showing the reactions that upon deletion impaired glucose secretion.

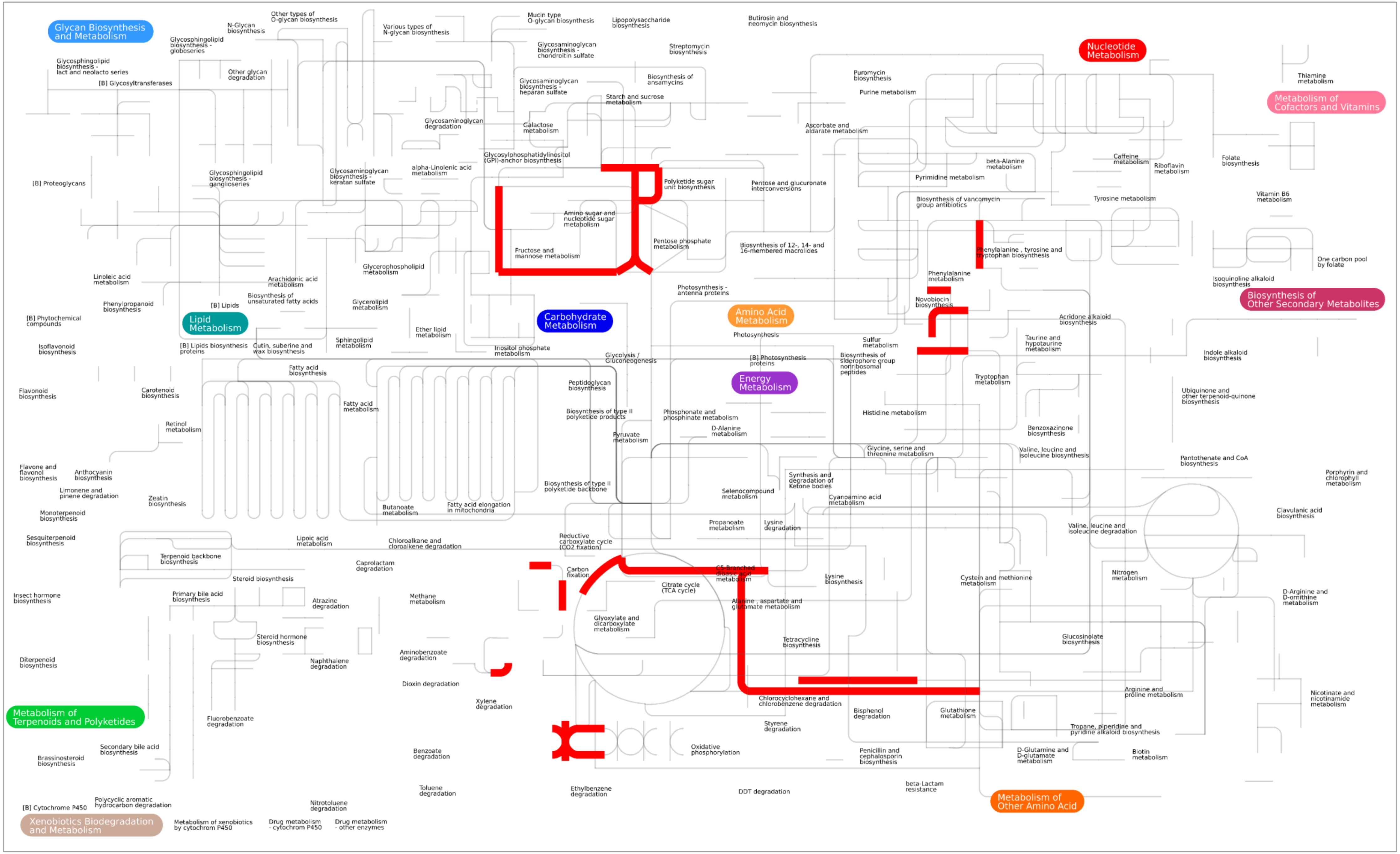

Supplementary figure S2. KEGG global metabolism map showing the reactions that upon deletion abolished TAG secretion.

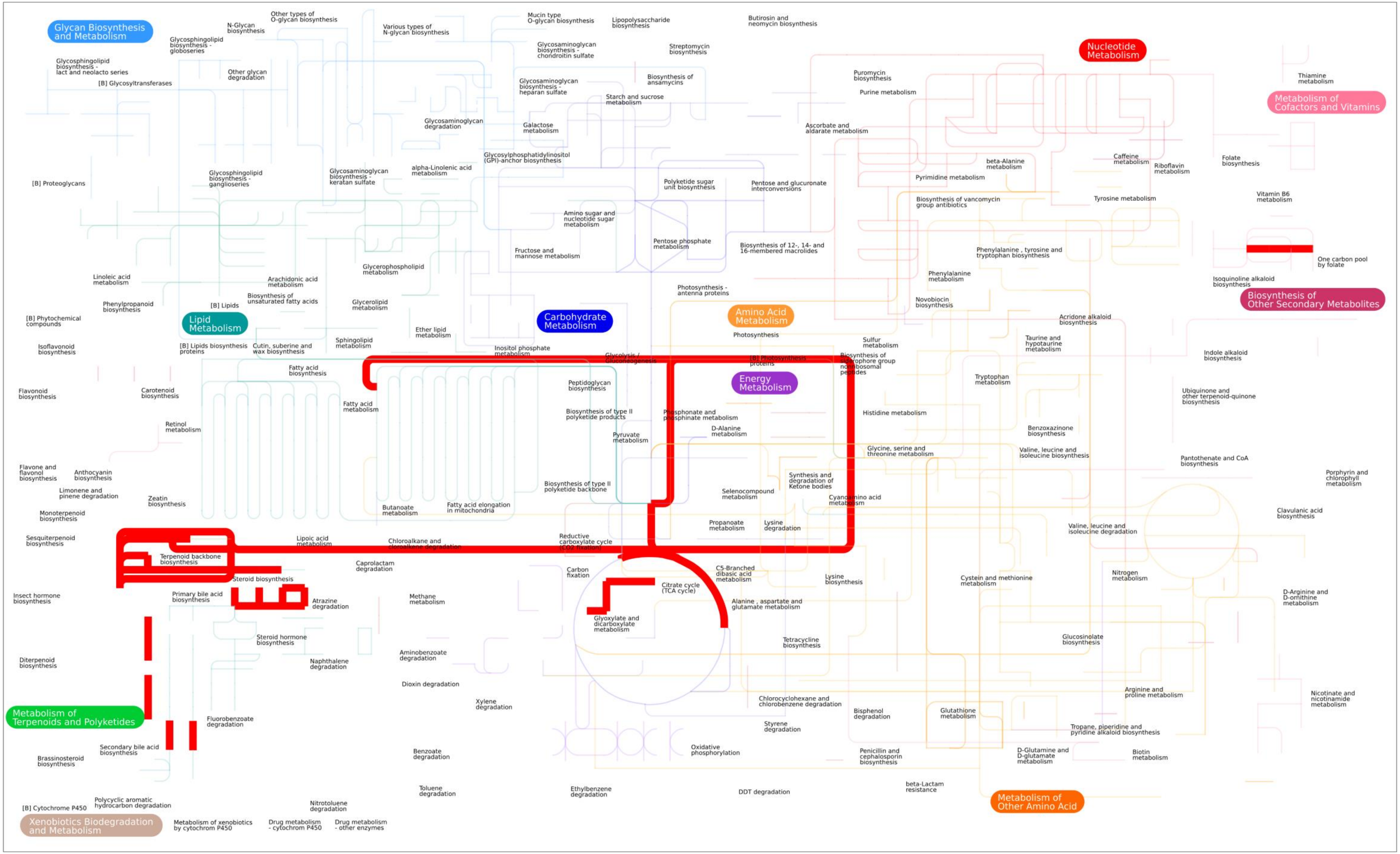

Supplement: Supplementary file 1 — Supplementary information [file 41598_2017_7350_MOESM1_ESM.pdf]
